# On the robustness of VMAT-SABR treatment plans against isocentre positioning uncertainties

**DOI:** 10.1186/1748-717X-9-196

**Published:** 2014-09-05

**Authors:** Joep Stroom, Sandra Vieira, Dalila Mateus, Carlo Greco, Antonella Fogliata, Giorgia Nicolini, Alessandro Clivio, Eugenio Vanetti, Luca Cozzi

**Affiliations:** Radiation Oncology Department, Medical Physics Unit, IOSI, Oncology Institute of Southern Switzerland, 6504 Bellinzona, Switzerland; Radiation Oncology Department, Champalimaud Foundation for the Unknown, Lisbon, Portugal

**Keywords:** RapidArc, SABR, Plan robustness, Acuros, AAA

## Abstract

**Background:**

To appraise the robustness of VMAT dose distributions against uncertainties in the positioning of the patients when single fraction SABRT treatments are planned.

**Methods:**

A set of 18 patients (8 lung, 5 brain, 5 spinal or para-spinal) treated with VMAT in a single fraction of 24Gy were retrospectively analyzed. All approved plans were re-calculated by applying shifts to the isocentre of ±0.5, ±1, ±1.5, ±2 and ±3 mm along the primary X, Y and Z axes. Dose calculations were performed with the AAA and the Acuros engines. Quantitative analysis of DVH was performed on a total of 36 references (18 patients with AAA, 18 with Acuros) and 1080 re-calculated plans to measure the potential degree of deterioration of the plans according to the simulated errors.

**Results:**

The dose to the CTV was essentially not affected by the isocenter shifts in all cases. Concerning PTV, The main impact was observed on the near-to-minimum dose D_99%_. No relevant impact was observed on organs at risk in the case of lung patients. In the case of patients treated in the spinal or para-spinal region, the near-to-maximum dose to the spine showed, in the worst scenario, referring to Acuros calculation, a potential average increase of 0.3Gy with a maximum of 1.9Gy (from 10.3 to 12.2 Gy) or 18%. This was partially mitigated to 12% with 1 mm and to 5% with 0.5 mm shifts.

**Conclusions:**

The study showed that shifts in the position of the isocenter as large as 3 mm tend to have modest impacts on the quality of the VMAT plans, scored by means of conventional DVH parameters. From the data shown, the VMAT approach should be considered adequately robust for single fraction SABR.

## Background

The recent introduction of volumetric modulated arc therapy (VMAT) in radiotherapy practice and the demonstration of its potential dosimetric benefit when compared to other techniques for intensity modulation has allowed to explore and revamp the role of stereotactic body radiotherapy in several indications. Preliminary encouraging results has led to more and more frequent application of extreme hypo-fractionation regimens, in the frame of the so-called stereotactic ablative radiation therapy (SABR) [1–9]. In particular, for the case of early stage or metastatic patients, single fraction SABR has been reported with promising clinical results. Filippi [10] reported about a cohort of 67 oligo-metastatic patients treated with single dose of 26Gy, where a 2-year local control of 88% was achieved. Palma [11] recommended to propose SABR to early stage patients also in the elderly, as data demonstrated that SABR improves the overall survival relative to the conventional radiation regimens.

While no definitive clinical data have been reported so far about long term outcome results, a number of studies were published about the investigation on safety aspects of the application of novel treatment modalities like VMAT in association with extreme hypo-fractionation. In particular, the potential interplay effect between intra-fractional patient movements (or mostly internal organ movement) and dynamic delivery patterns was appraised. At a first level of complexity, Ong [12] studied the interplay effect of VMAT in stereotactic lung treatments with conventional flattened beams. The experiments allowed to conclude that VMAT, in its RapidArc (RA) form, delivered accurately the calculated dose and that the tumor motion did not significantly deteriorated the dose distribution for single-fraction treatments when two arcs were applied. Similar results were obtained by Stambaugh [13] also in the presence of significant tumor motion (excursions of the target as great as 2–3 cm). The complexity of the interplay assessment increased when high intensity photon beams (the flattening filter-free beams, FFF) were introduced in the clinics. With these beams, dose rates as great as 2400 MU/minute could be generated and delivered to the patients suggesting a potentially increased risk. Ong [14, 15] studied the case of lung and spinal irradiations with RA and FFF beams and recognized that the interplay might be present but mitigated when multiple arcs were applied. Peguret [16] quantified the intrafraction lung tumor position motion during high dose rate SABR and concluded that, from the analysis of a cohort of 32 patients and 140 treatment fractions, the mean vector of the tumor motion was about 2 mm. Li [17] further refined the analysis by investigating the dosimetric impact of the intra-fractional motion by using intrafraction cone beam CT data acquired during lung SABR. Authors concluded that the GTV was consistently covered by the prescription dose for all patients despite of some blurring in the dose distributions (imputable to imperfect breath-hold reproducibility or residual GTV motion).

The role and the risks associated to intra-fraction motion seems to be relatively well understood and data suggests a substantial robustness of VMAT, also with FFF beams. Nevertheless, studies in homogeneous phantoms never reflects the dose effect in complex clinical cases and, due to the risk of uncontrolled potential significant errors, all departments should investigate the interplay effect with their own equipments and methods. The SBRT/SABR treatments with VMAT (but not only) could be prone also to other sources of errors. In particular the precision of the patient positioning, or in other terms the precision of the treatment isocentre positioning with respect to the planned one. A limited accuracy could introduce some kind of “baseline” shift with significant dosimetric consequences.

Dahele [18] analyzed lung patient positioning during SBRT in patients treated without rigid immobilization systems. The study, based on a cohort of 109 SBRT fractions, quantified that, over a mean treatment time of about 10 minutes (with 4 minutes of beam on time), the mean motion vector of the target centroid position was smaller than 1 mm, and <1.7 mm in the vast majority of the cases. This means that, if properly positioned, there is a minimum risk of time depending drift of the target when fast VMAT is applied. This drift would superimpose to the intra-fractional motion discussed above.

Little is known and today published about the basic question of VMAT plan robustness to the initial positioning precision. Some investigations have been performed to introduce optimization tools that could increase the plan robustness in intensity modulation therapy. An interesting approach has been explored but Baum et al. [19] introducing the concept of coverage probabilities in the definition of the cost functions and the local weights in the optimization phase. From a different perspective, Zhang et al. [20], proposed to incorporate treatment outcome knowledge to shape and adapt the dose distributions to the probability of residual disease via a predictive method. These highly promising conceptual approaches, very different in the aims but all concurring in the optimization of stronger plans, have hardly been explored in routine and none is available in standard treatment planning systems.

Aim of the present study was to investigate the robustness of SABR plans optimized with the RA technique and FFF beams to various levels of positioning misplacements and to determine if the photon calculation algorithm used for the dose calculation can magnify or mitigate the potential risk of under-treatment of the patients.

## Materials and methods

For the study, a set of 18 patients was prepared. This included 8 patients treated for lung tumors, 5 patients treated for brain metastases and 5 for spinal or para-spinal metastases. All patients were treated with a single fraction SABR and a prescription dose of 24Gy (optimized with the requirement that for the planning target volume PTV, V_24Gy_ > 99%). Plans were optimized using the RapidArc technique on a TrueBeam-STX linac equipped with a high definition multileaf collimator (with 2.5 mm spatial resolution at isocentre in the inner part). 6MV or 10MV FFF beams were used for all the plans and the jaw tracking option activated in the optimization. In all cases selected for the study, the isocentre was located in the target volume. For the lung patients, the margin between CTV and PTV was between 3 to 5 mm, for the brain patients this was 3 mm and for the spinal patients it was 2 mm.

The study was performed using two different photon dose calculation algorithms implemented in the Eclipse treatment planning system: the Anisotropic Analytical Algorithm (AAA) and the Acuros-XB (AXB) [21–24] for both algorithms the version 13.0 was used. For all patients two reference plans were computed (one per algorithm) using the same optimization as an input for the final dose calculation. To assess the robustness of the plans against simulated isocentre displacements a new tool implemented in the version 13 of Eclipse was used. This tool, called ‘Plan Uncertainty’, allows to semi-automatically generate, calculate, and save dose distributions of replica-plans. In these replica-plans arbitrary shifts of the isocentre can be applied along the X, Y (axial) or Z (cranio-caudal) directions. The original plan was used for all the re-calculations. For the study it was chosen to apply shifts on one axis at the time only, to apply both positive and negative shifts (with respect to the reference isocentre coordinate) and to simulate 5 groups of shifted plans by applying 0.5, 1, 1.5, 2 and 3 mm shifts. These isocentre shifts have been chosen to mimic high (<1 mm), medium (<2 mm) and low (>2 mm) precision in the patient’s positioning before treatment. No assumptions have been made to correlate the possible precision to the image guidance and immobilization tools applied in clinical practice, this being out of the investigational scope.

In total 30 replica-plans were generated per patient and algorithm, resulting in a total of 36 reference plans and 1080 replica-plans affected by isocentre shifts.

For all these plans, the dose volume histograms (DVH) of the target volumes (CTV and PTV) and of the main relevant organs at risk per each case were exported from Eclipse and analyzed with a dedicated home-grown software. For the lung patients, the ipsi-lateral lung was chosen as the main critical organ. For the brain patients the brainstem, and for the spinal cases the spinal cord.

The analysis performed on all the DVH was stratified according to the algorithm, the isocentre shift and, obviously, the patients group. Conventional dose-volume parameters were computed from the DVH to study target coverage (near-to-minimum doses as D_98%_ and D_99%_), target homogeneity (D_5%_-D_95%_) and mean target dose. For the organs at risk, the mean and/or the near-to-maximum (D_1%_, D_2%_) doses were investigated.

The lung cases (planned on an average CT from a 4D acquisition) were chosen to represent situations where the RA plan robustness was assessed against positioning errors potentially shifting the isocentre from soft-like tissue to lung-like tissues. The spinal cases were chosen to investigate the situation where the shifts might move the isocentre from the bone-like to soft-tissue-like tissues; finally, the brain cases were select to appraise the case where the isocentre would likely remain inside soft-tissue irrespective to the applied shift. With these three categories it is possible to determine the interplay between photon dose calculation algorithms and SABR RA plans robustness to patient positioning errors.

## Results

**Figure 1:**
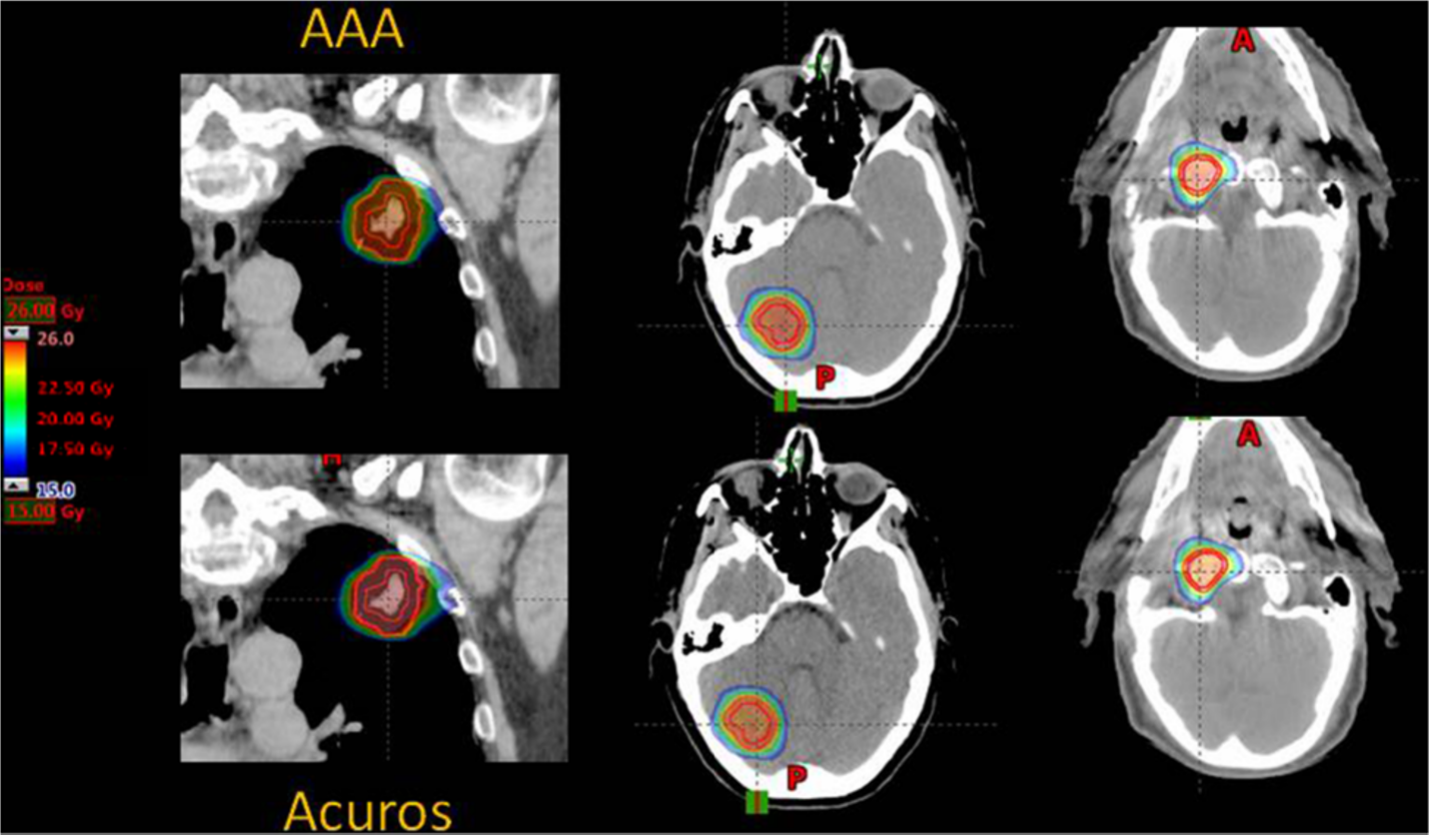
**Example of dose distribution in an axial view for a lung, brain and spine patient.** Data are relative the two reference plans. Color wash is set between 15 and 26 Gy.

Figure 1 shows, for one example lung, brain and spinal patients, the dose distribution for the reference plans calculated with either the AAA or the AXB algorithms.

**Figure 2:**
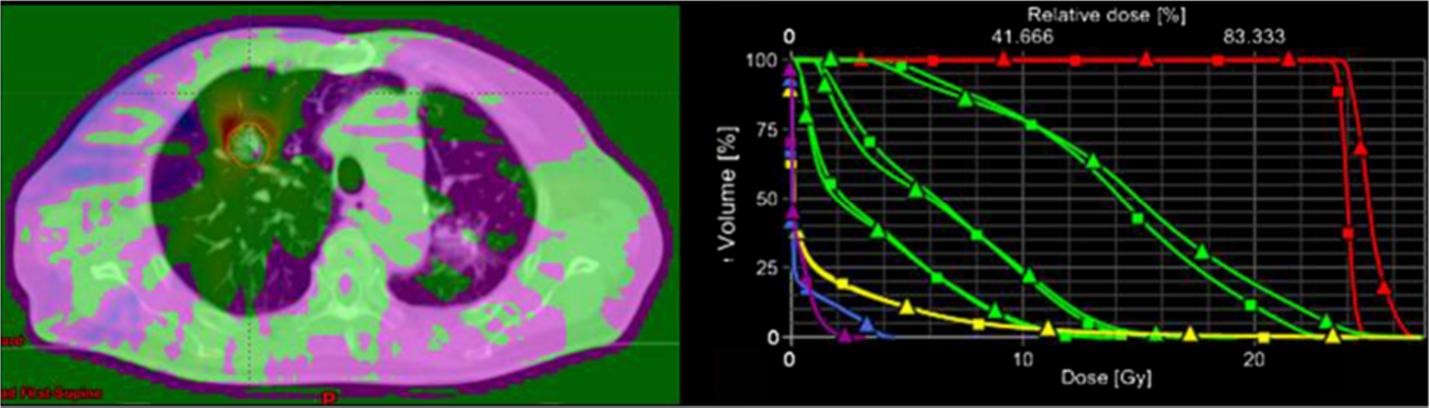
**The potential bias effect between AXB and AAA in the dose distributions when lung tissue is involved.** In the left panel an axial view of the dose difference between AAA and AXB dose distributions and in the right panel the DVH display for the two plans. In red the PTVs, in green the ipsilateral lung, the ipsilateral lung minus the PTV and the contralateral lung, in yellow the body, in blue the esophagus and in magenta the spinal cord. Triangles correspond to Acuros, the squares to AAA.

Figure 2 shows the potential bias effect between the usage of AXB (triangles) or AAA (squares) in the dose calculation when lung tissue is involved. DVHs are shown for the PTV and various organs at risk (ipsi- and contra-lateral lungs, heart, spinal cord, esophagus, trachea). Depending upon the normalization strategy, the graph suggest the potential target mis-dosage that occurs in patients when calculation is performed on AAA (V24Gy drops from 98% to ~45%). This issue is not part of the present study.

**Figure 3:**
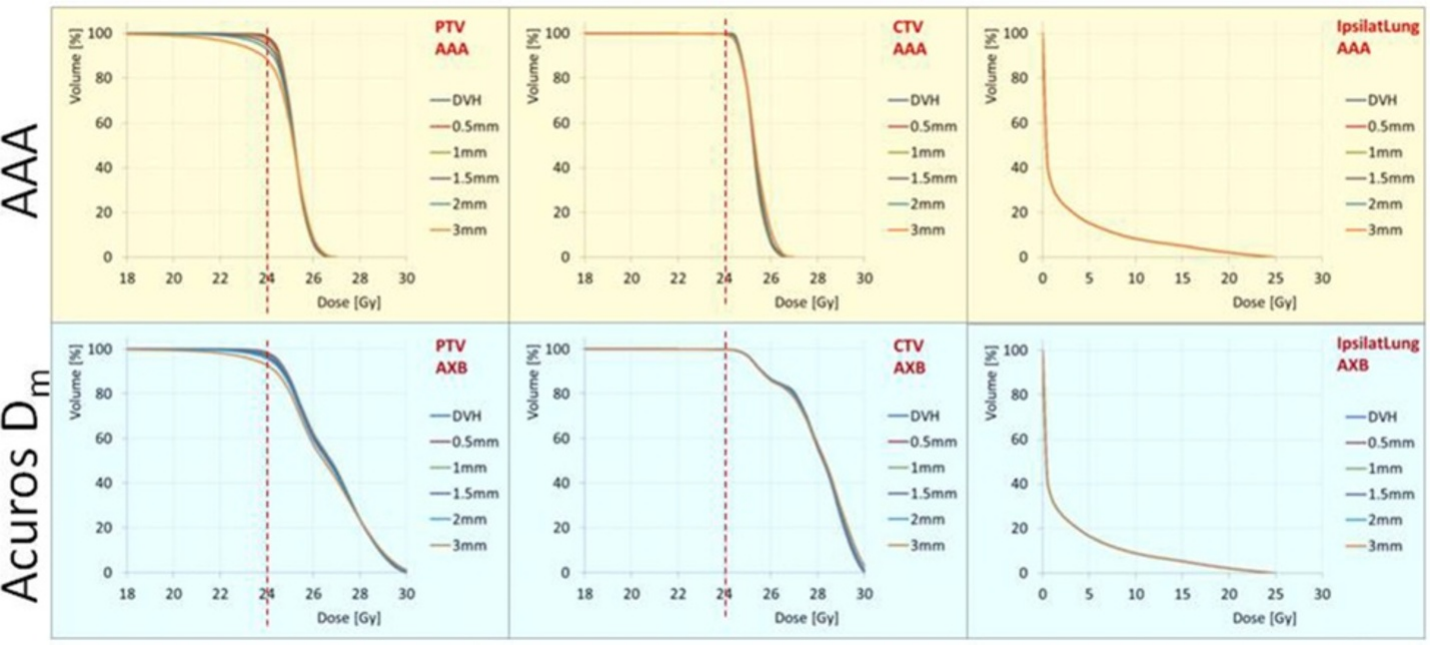
**The average dose volume histograms over the lung patients cohort for the reference plans and the plans recalculated after the application of the various isocenter shifts.** Each of these curve in the graphs corresponds also to the average over the 6 plans recalculated for each of the shifts (±X, ±Y and ± Z). The results are shown for the PTV, the CTV and the ipsilateral lung.

Figure 3 shows the average DVH over the lung patients cohort for the reference plans and the plans recalculated after the application of the various isocenter shifts. Each of the curves in the graphs corresponds also to the average over the 6 plans recalculated for each of the shifts (±X, ±Y and ± Z). The results are shown for the PTV, the CTV and the ipsilateral lung.

**Figure 4:**
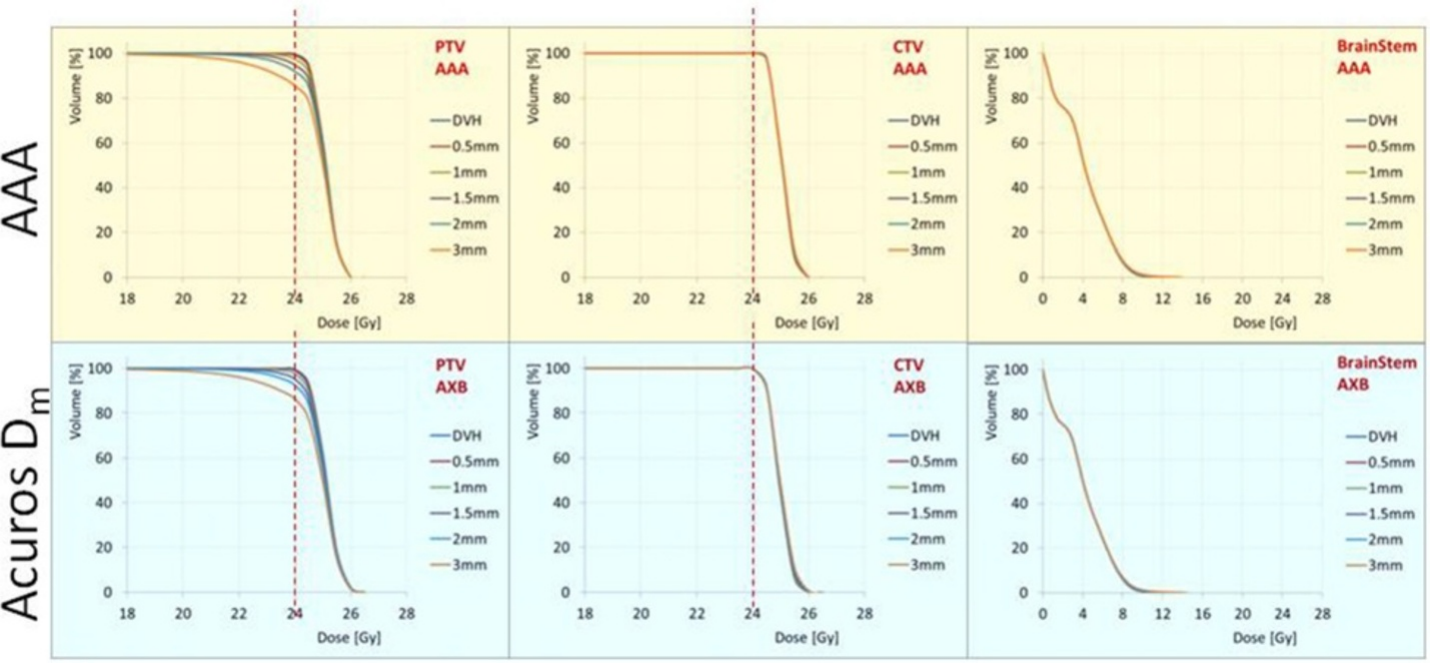
**The average dose volume histograms over the brain patients cohort for the reference plans and the plans recalculated after the application of the various isocenter shifts.** Each of these curve in the graphs corresponds also to the average over the 6 plans recalculated for each of the shifts (±X, ±Y and ± Z). The results are shown for the PTV, the CTV and the brain stem.

**Figure 5:**
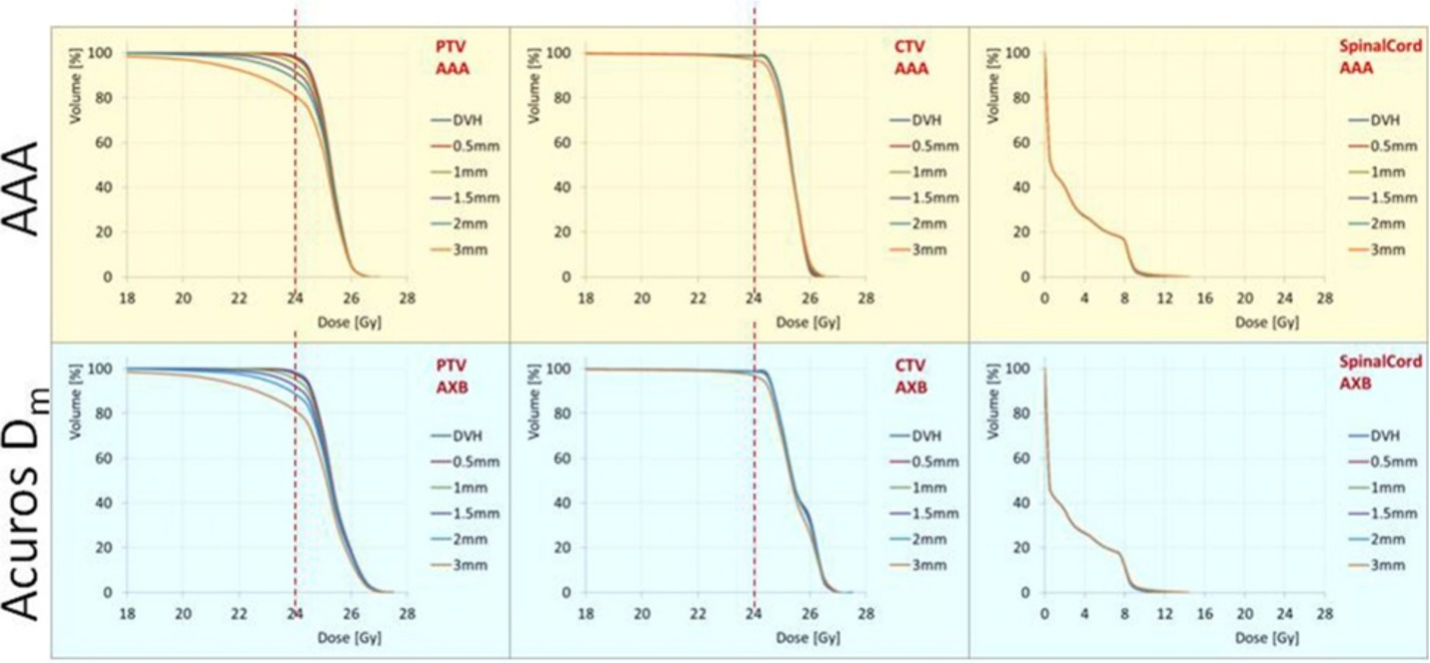
**The average dose volume histograms over the spinal patients cohort for the reference plans and the plans recalculated after the application of the various isocenter shifts.** Each of these curve in the graphs corresponds also to the average over the 6 plans recalculated for each of the shifts (±X, ±Y and ± Z). The results are shown for the PTV, the CTV and the spinal cord.

Figures 4 and 5 show the same results in the cases of brain and spinal patients. In these cases, the relevant organs at risk were the brain stem and the spinal cord, respectively.

**Figure 6:**
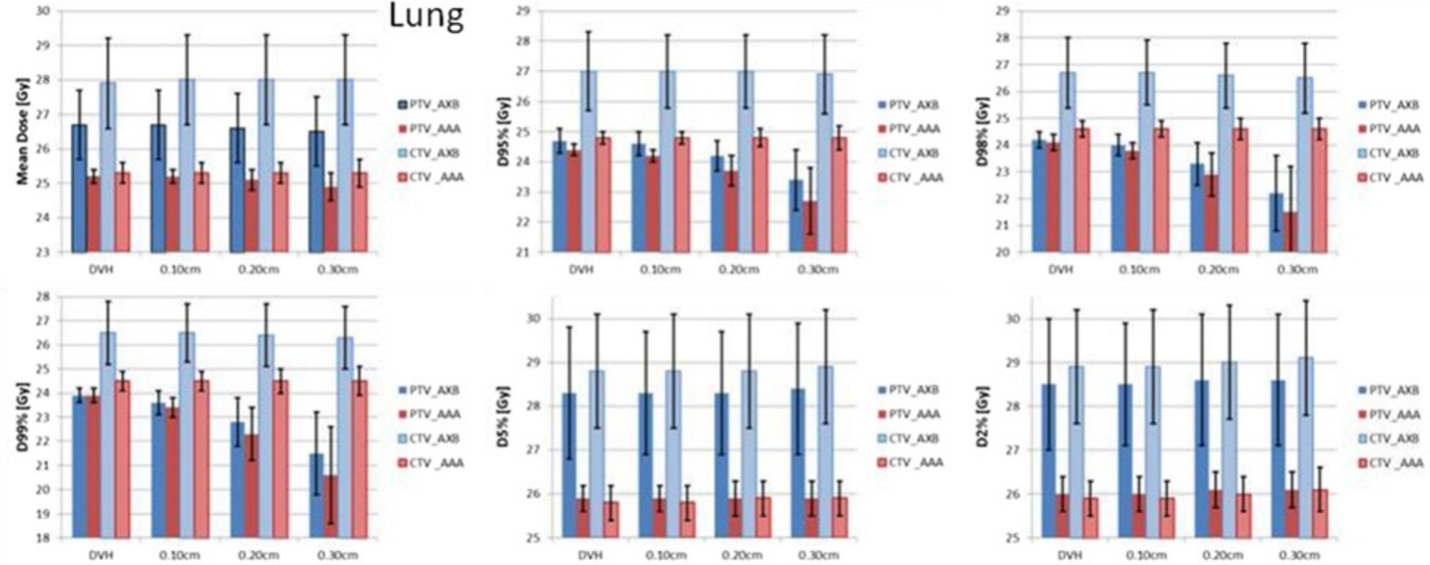
**The summary of the quantitative analysis on the DVH performed for all the lung patients and the various shifts.** Results are shown for the mean dose, D_95%_, D_98%_, D_99%_, D_5%_ and D_2%_ and are stratified for the PTV and the CTV and for the AXB or AAA calculations.

Figure 6 shows the summary of the quantitative analysis on the target DVHs performed for all the patients and the various shifts. Error bars are computed over the patients and the shifts and are reported at 1 standard deviation. Results are shown for the mean dose, D_99%_, D_95%_ and D_5%_, D_98%_ and D_2%_, and are stratified for the PTV and the CTV and for the AXB or AAA calculations.

**Figure 7:**
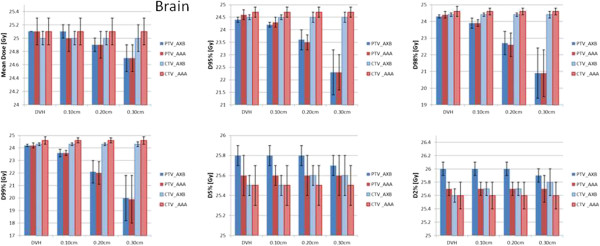
**The summary of the quantitative analysis on the DVH performed for all the brain patients and the various shifts.** Results are shown for the mean dose, D_95%_, D_98%_, D_99%_, D_5%_ and D_2%_ and are stratified for the PTV and the CTV and for the AXB or AAA calculations.

**Figure 8:**
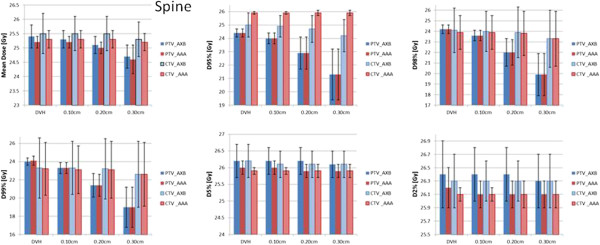
**The summary of the quantitative analysis on the DVH performed for all the spine patients and the various shifts.** Results are shown for the mean dose, D_95%_, D_98%_, D_99%_, D_5%_ and D_2%_ and are stratified for the PTV and the CTV and for the AXB or AAA calculations.

Figures 7 and 8 represent the same results for the brain cases and the spinal cases. Table 1 shows the conformity index CI95% (defined as the ratio between the volume of the 95% isodose and the target volume) for the baseline and the various shifts (averaged over the patients). The conformality of the dose distributions resulted un-affected by the various shifts.

**Table 1:** **Conformity index (CI95%)**

				Lung			
	**0.0 [mm]**	**0.5 [mm]**	**1 [mm]**	**1.5 [mm]**	**2 [mm]**	**3 [mm]**	**p (fischer)**
** AAA**	2.1 ± 1.0	2.1 ± 0.9	2.1 ± 0.9	2.1 ± 0.9	2.1 ± 0.9	2.1 ± 0.9	n.s.
**Acuros**	1.8 ± 0.6	1.8 ± 0.6	1.8 ± 0.6	1.8 ± 0.6	1.8 ± 0.6	1.8 ± 0.6	n.s.
				**Brain**			
** AAA**	2.7 ± 0.6	2.7 ± 0.5	2.7 ± 0.5	2.7 ± 0.5	2.7 ± 0.5	2.7 ± 0.5	n.s.
**Acuros**	2.2 ± 0.8	2.2 ± 0.6	2.2 ± 0.6	2.2 ± 0.6	2.2 ± 0.6	2.2 ± 0.6	n.s.
				**Spine**			
** AAA**	3.6 ± 1.8	3.6 ± 1.5	3.6 ± 1.5	3.6 ± 1.5	3.6 ± 1.5	3.6 ± 1.5	n.s.
**Acuros**	2.1 ± 0.9	2.1 ± 0.8	2.1 ± 0.8	2.1 ± 0.8	2.1 ± 0.8	2.1 ± 0.8	n.s.

Results are shown for the three groups and two algorithms and the various isocentre shifts.

In the case of lung, the mean dose results are higher with AXB then with AAA and the dose difference is statistically significant in all cases. The CTV mean dose results are higher than the mean dose to PTV and it scales also according to the algorithm used. The target heterogeneity (D_5%_-D_95%_) is greater with AXB and the near-to-minimum dose (D_98%_ and D_99%_) is higher.

When considering the isocenter shifts, the impact on CTV and ipsilateral lung results appears to be modest for both the algorithms and for all the applied shifts. On the contrary, the PTV coverage shows a clear trend to deterioration with increasing shifts, more remarkable with AAA. V_24Gy_ drops from 98.6 ± 1.3% and 98.4 ± 1.0% for the reference plans to 88.4 ± 5.2 and 92.8 ± 3.5% for the 3 mm shift for AAA and AXB, respectively. The corresponding values for CTV remains greater than 99.5% in all cases.

In the case of spinal treatments, the PTV coverage was compromised with all shifts and remarkably above 1 mm for both algorithms. For the CTV only misplacements as great as 3 mm resulted in visible effects reducing the minimum dose below 99% (as expected with a margin of 2 mm).

The data demonstrate the estimated mean dose results only slightly greater with AXB compared to AAA (with a difference <1% in average). The target dose heterogeneity increases modestly with increasing isocentre misplacement and it is similar for both algorithms. The near-to-minimum doses (D_98%_ and D_99%_) are similar between algorithms and show for the PTV a marked reduction above 2 mm while it remains modest for the CTV. V_24Gy_ = 98.5 ± 2.5% and 98.3 ± 2.4% for AAA and AXB, respectively are for the reference plans, while these values drop to 80.9 ± 7.7 and 81.2 ± 7.3% for the 3 mm shift. At 2 mm, V_24Gy_ = 88.3 ± 5.1% and 88.7 ± 4.8% for AAA and AXB, respectively. The corresponding values for CTV remains greater than 99.5% in all cases except for the 3 mm shift (98%). The spinal cord demonstrated an increase of the maximum point dose with the increased isocentre misplacement but not at the level of D_2%_.

The analysis of the brain cases revealed similar effects as the above but in this case, the estimated mean PTV dose did not differ between algorithms as well as the target dose inhomogeneity and the minimum dose. The PTV coverage was compromised significantly for misplacements greater than 1 mm and macroscopically for 3 mm. The trends were very similar between AAA and AXB. Negligible effects were observed for the CTV and the brainstem D_1%_ (while the point dose increases with increasing mispositioning), irrespective to the calculation algorithm. V_24Gy_ = 99.6 ± 0.4% and 99.5 ± 0.3% for AAA and AXB, respectively are for the reference plans while these values drop to 85.6 ± 2.5% and 86.3 ± 3.2% for the 3 mm shift. At 2 mm, V_24Gy_ = 91.9 ± 1.8% and 92.5 ± 2.4% for AAA and AXB, respectively. The corresponding values for CTV remains greater than 99.8% in all cases.

## Discussion

From all these data, it is possible to share some considerations about the issue of plan robustness to isocenter misplacements for VMAT-based SABR treatments.

In general, with the CTV to PTV margins applied in the study (5 mm for the lung, 3 mm for the brain and 2 mm for the spine), the coverage of the clinical target volume is not compromised in most of the cases even for very large (and unlikely) positioning errors. In particular this applies to the lung patients, where the potential of errors might be greater, and in the brain. An intermediate situation was observed for the spine (errors of 3 mm might have some impact on the CTV coverage). Although in the case of the spine the CTV to PTV margin is partially in the bone and partially in soft tissue and in case of the lung the margin is mostly in lung tissue, the more accurate AXB calculation does not reveal subtle risks of increased under-coverage. On the contrary, the isocentre positioning precision plays a relevant role in the case of the PTV and the data demonstrate that when the misplacement is comparable to the CTV to PTV margin, then the under-dosage estimation on the PTV becomes very large. The intensity of the effect depends on the algorithm applied. In fact, the 10% reduction in V_24Gy_ of the PTV for the AAA data is reduced to about 5.5% with AXB.

In summary, the AAA algorithm seems to be adequate for the assessment of the CTV dose, while the use of AXB might be preferable for a better appraisal of the PTV coverage. VMAT plans appear to be more robust to uncertainty in the isocenter positioning when using the more accurate algorithm where the lateral scattering is accounted for. Clinically, the relevant question is whether the proven robustness for the CTV is sufficient or, depending on the lung tissue composition, the actual tumor spread and the density of clonogenic cells, the potential underdosage in the PTV region should be known (and controlled) with the highest accuracy. In the working hypothesis that all the microscopic disease is propely included in the CTV (as per theoretical definition), this should be sufficient. In this case, AXB and an isocentre positioning better than 2 mm would be needed. In addition, the data from Peguret [16] showed that the intra-fraction change in tumor position in lung patients could be of the order of 2 mm which would further magnify the relevance of precise positioning at the level of 1 mm.

Plan robustness analysis in the case of the spinal treatments lead to some interesting finding. Despite the CTV being mostly located in bony structures while the margin to the PTV in soft tissues, the differences observed between the algorithms are practically irrelevant, showing a counter-intuitive limited importance of the dose-to-water vs. dose-to-medium calculation approach. The relevant effect in terms of robustness is given only by the magnitude of the isocentre displacement with respect to the reference and with respect to the margins applied. Above 1–2 mm the risk of mis-treatment seems to be relevant.

The apparently simpler clinical case was the group of brain patients. In these plans, the targets are located in a “bath”. As a consequence, confirmed by the data, the role of the calculation algorithm is negligible. On the contrary, given the tight dose gradients generated by the VMAT plans, isocentre positioning precision should be granted at the level of 1 mm or better to guarantee full PTV coverage.

## Conclusion

A cohort of RapidArc based SABR plans were analyzed in terms of their dosimetric robustness against compromised precision in the positioning of the treatment with respect to the planned isocentre. Results demonstrated that for low and medium errors in the isocentre position (i.e. typically up to 2 mm errors) the coverage of the CTV is not compromised while possible relevant errors can be observed for the PTV. Use of AXB can mitigate the intensity of the effect. Use of very tight margins between CTV and PTV should be carefully evaluated and correlated with the degree of precision achievable in the entire patient positioning procedure.

The most relevant parameter to ensure plan robustness in RapidArc based SABR is the appropriate use of margins between the tumor volume (CTV or GTV) and the PTV.
